# Green synthesis of spiro[indoline-3,4′-pyrano[2,3-c]pyrazoles] using Fe_3_O_4_@l-arginine as a robust and reusable catalyst

**DOI:** 10.1186/s13065-019-0636-1

**Published:** 2019-10-11

**Authors:** Mohammad Ali Ghasemzadeh, Boshra Mirhosseini-Eshkevari, Mohammad Hossein Abdollahi-Basir

**Affiliations:** 1 Department of Chemistry, Qom Branch, Islamic Azad University, Post Box: 37491-13191, Qom, Islamic Republic of Iran; 2 Department of Chemistry, Faculty of Sciences, University of Guilan, Rasht, Islamic Republic of Iran

**Keywords:** Multi-component reactions, Fe_3_O_4_@l-arginine, Nanocomposite, Spiropyranopyrazoles, Amino acid, Catalyst

## Abstract

The synthesized Fe_3_O_4_@l-arginine showed strong catalytic performance in the one-pot synthesis of spiropyranopyrazoles via the reactions of hydrazines, β-keto esters, isatins, and malononitrile or ethyl cyanoacetate under solvent-free conditions. The biologically active heterocyclic compounds including spiropyranopyrazole derivatives were efficiently synthesized in short reaction times and excellent yields in the presence of Fe_3_O_4_/l-arginine at room temperature. The highlighted features of the Fe_3_O_4_@l-arginine nanocomposite are highly stable, easy to separate, low loading, cost-effective with easy preparation and reusability of the catalyst. The heterogeneous nanocomposite was fully characterized by SEM, EDX, FT-IR, XRD and TEM analysis.

## Introduction

One-pot multi-component reactions (MCRs), is an interesting synthetic strategy for the synthesis of small-molecule libraries with various degrees of structural variety because various organic moieties are joined in one step for carbon–carbon and carbon-heteroatom bond formation [1, 2]. They offer considerable advantages over ordinary linear step synthesis by decreasing time, saving money, energy, and crude materials. Therefore, they result in both economical and environmental benefits. At the same time, variety can be gained from building up libraries by differing each component [3, 4]. In recent years, there has been massive development in three- and four-component reactions, and great efforts continue to be made to expand new MCRs [5]. Spirocyclic compounds are considered as significant building canton for the easy availability of a diversity of cyclic products by a sequential reaction due to their steric strain attendant with the quaternary carbon [6]. Expansion of new procedures for producing spirocyclic compounds is an interesting and challenging function in organic synthesis [7]. One of the significant multi-component reactions is the synthesis of spiropyranopyrazole derivatives which exhibit high medicinal attributes and biological activities. Spiropyranopyrazoles area class of nitrogen heterocyclic compounds with considerable and well- offered biological activities that consist of antimicrobial [8], anti-inflammatory [9], anticancer [10] and molluscicidal activities [11]. Spiropyranopyrazoles are important heterocyclic compounds due to their variety and pharmaceutical biological activities [12]. Concerning the arithmetic of the significance of oxindole parts in organic compounds, as well as the intrinsic complexity of isatins as heterocyclic substrates, it is not amazing that many diverse and elegant MCRs have been introduced for the synthesis of various heterocyclic and spiroheterocyclic products by using isatins as a core component [13]. Therefore, different synthetic approaches for the synthesis of spirooxindole-fused heterocycles have been reported and reviewed [14]. Previous studies have described procedures to synthesize of pyrano[2,3-c]pyrazoles using several catalysts such as cerium ammonium nitrate [15], l-proline [16], piperidine [17] and cobalt NPs [18].

Nevertheless, there are only two reported methods in the literature for the synthesis of spiro[indoline-3,4′-pyrano[2,3-c]pyrazole] derivatives via four-component reaction of hydrazines, ethyl acetoacetate, isatins, and malononitrile or ethyl cyanoacetate which have been done in the presence of piperidine [19], and Et_3_N [20].

Magnetic organic–inorganic nanocomposites have recently been the subject of intense research as magnetic catalysts in both industrial and academic settings. These magnetic nanoparticle catalysts can be used for investigating the reusing and seclusion problems that occur in several homogenous and heterogeneous catalytic reactions. Supported magnetic metal nanoparticles as new class of nanocatalysts have received much attention in diverse fields. The main feature of these particles is their high surface area that leads to their higher catalytic activity in comparison with traditional heterogeneous acid catalysts [21–27].

One of the outstanding procedures for preventing particle aggregation is coating nanoparticles with various targeting factors, taking into account their biocompatibility. Among the chemicals that can be used for achieving this target, amino acids are appropriate because of their crucial role in the body [28]. Amino acids react with the nanoparticles’ surface via the carboxyl groups and side chains [29]. Amino-functionalized materials demonstrate excellent ability to remove an extensive range of heavy metal ions from aqueous solutions because of the potent affinity between the nitrogen atom and metal cations [30]. Among different nanocomposite, Fe_3_O_4_/amino acid has received great attention in different fields because of their unique attributes and potential functions [31]. Some crucial characteristics of these catalysts include high catalytic activity, facile separation through an external magnet with no need for filtration, eco-friendliness, and non-toxicity. Recently, functionalized magnetic nanoparticles have been utilized as a useful catalytic system in numerous chemical processes such as synthesis of α-amino nitriles [32], *bis*(indolyl)methane derivatives [33], indazolo[2,1-b]phthalazine-triones and pyrazolo[1,2-b]phthalazine-diones [34], 3,4-dihydropyrimidin-2(1*H*)-ones [35], 1,8-dioxo-octa hydro xanthene derivatives [36], 2-amino-4*H*-chromen-4-yl phosphonates [37], 1,4-dihydropyridines [38] and pyrrole synthesis [39].

**Fig. 1:**
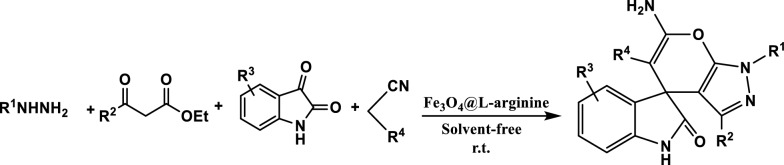
Synthesis of spiro[indoline-3,4′-pyrano[2,3-c]pyrazoles] using Fe_3_O_4_@l-arginine nanocomposite under solvent-free conditions

In continuation of our study in the synthesis of heterocyclic compounds using heterogeneous nanostructures [40–44], herein we describe a highly efficient and straightforward method for the synthesis of spiro[indoline-3,4′-pyrano[2,3-c]pyrazoles] via multi-component reaction of hydrazines, β-keto esters, isatin and malononitrile or ethyl cyanoacetate using Fe_3_O_4_@l-arginine as a green, economic, available and environmentally benign nanocatalyst under solvent-free conditions (Fig. 1).

## Results

### Catalyst characterization

In the preliminary experiments Fe_3_O_4_@l-arginine nanoparticles were prepared and characterized by SEM, EDX, FT-IR and XRD spectroscopy tenchniques.

**Fig. 2:**
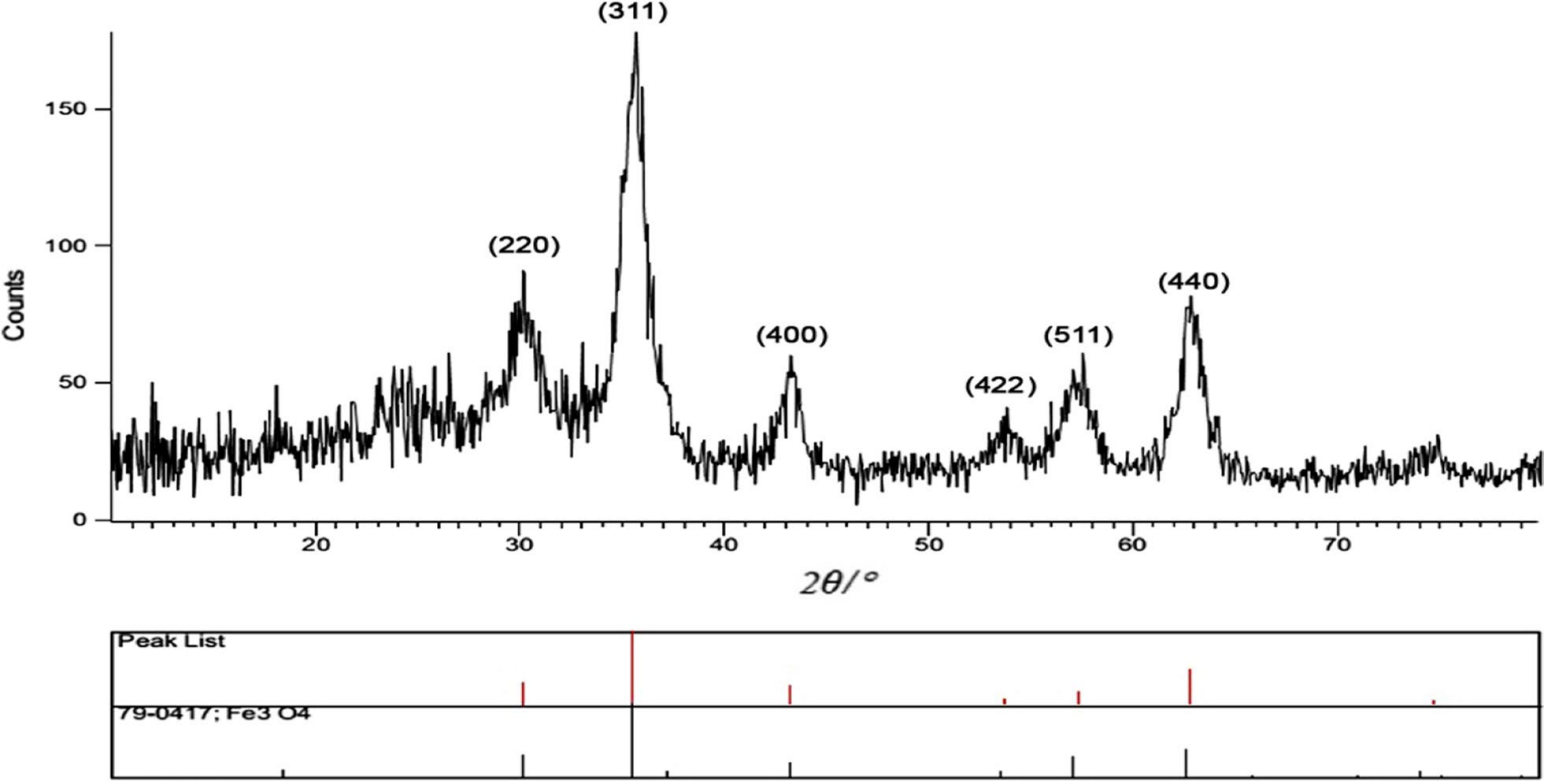
The X-ray diffraction pattern of the Fe_3_O_4_@l-arginine nanocomposite

The earned lattice parameter of the nanoparticle Fe_3_O_4_@l-arginine using XRD technique coincided to the standard parameters of magnetite. The pattern of the Fe_3_O_4_@l-arginine nanocomposite is depicted in Fig. 2. It could be seen that the strong diffraction peaks at 2θ of 30.1°, 35.4°, 43.2°, 53.7°, 56.9° and 62.9° belong to the peaks of (220), (311), (400), (422), (511) and (440) of the Fe_3_O_4_, which is similar to the bare Fe_3_O_4_ nanoparticles [40, 45].

**Fig. 3:**
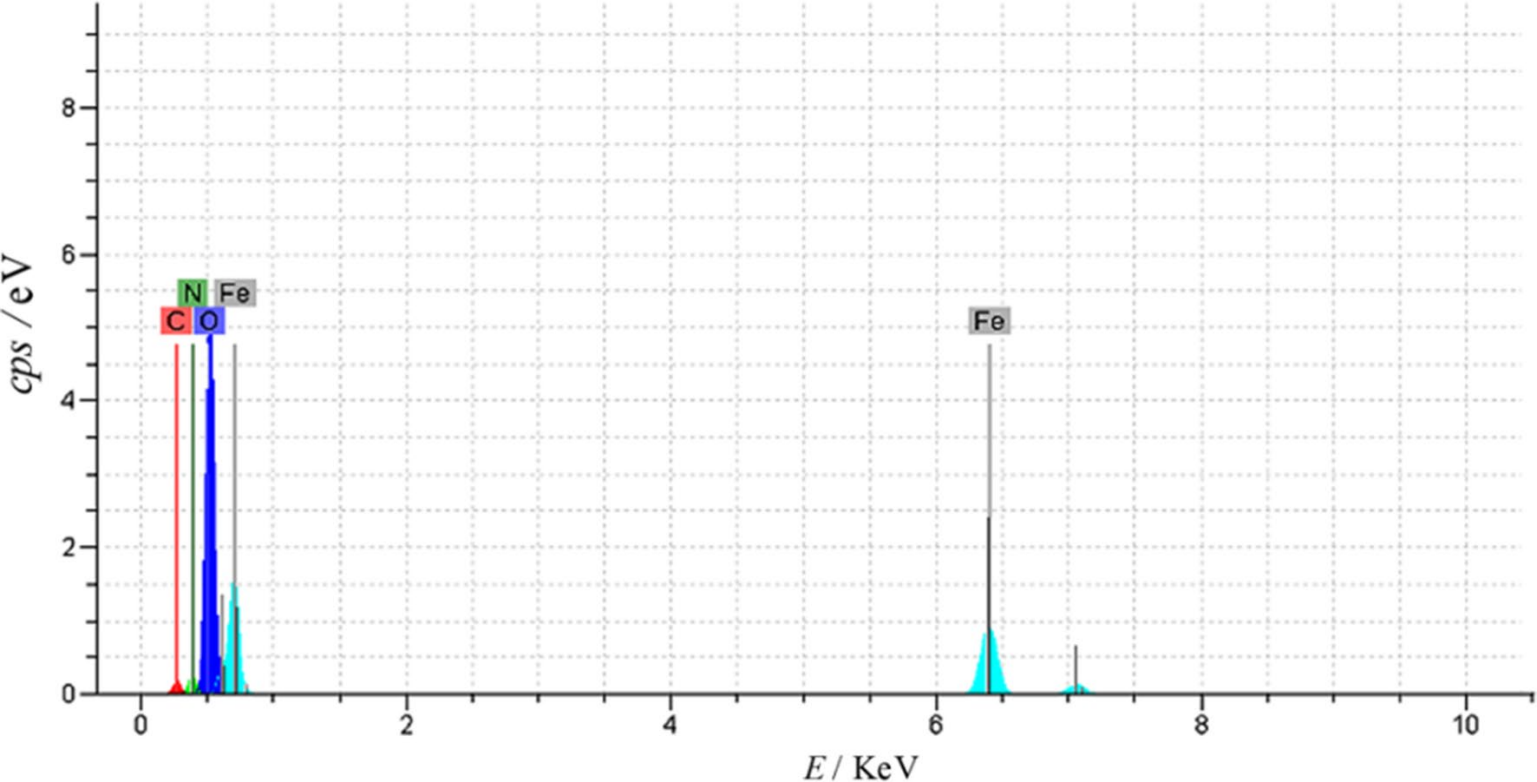
EDX spectrum of the Fe_3_O_4_@l-arginine nanocomposite

The chemical purity of the sample, as well as their stoichiometry, was tested by EDX study. Figure 3 shows that the elemental compositions of Fe_3_O_4_@l-arginine are Fe, O, C, H, and N.

**Fig. 4:**
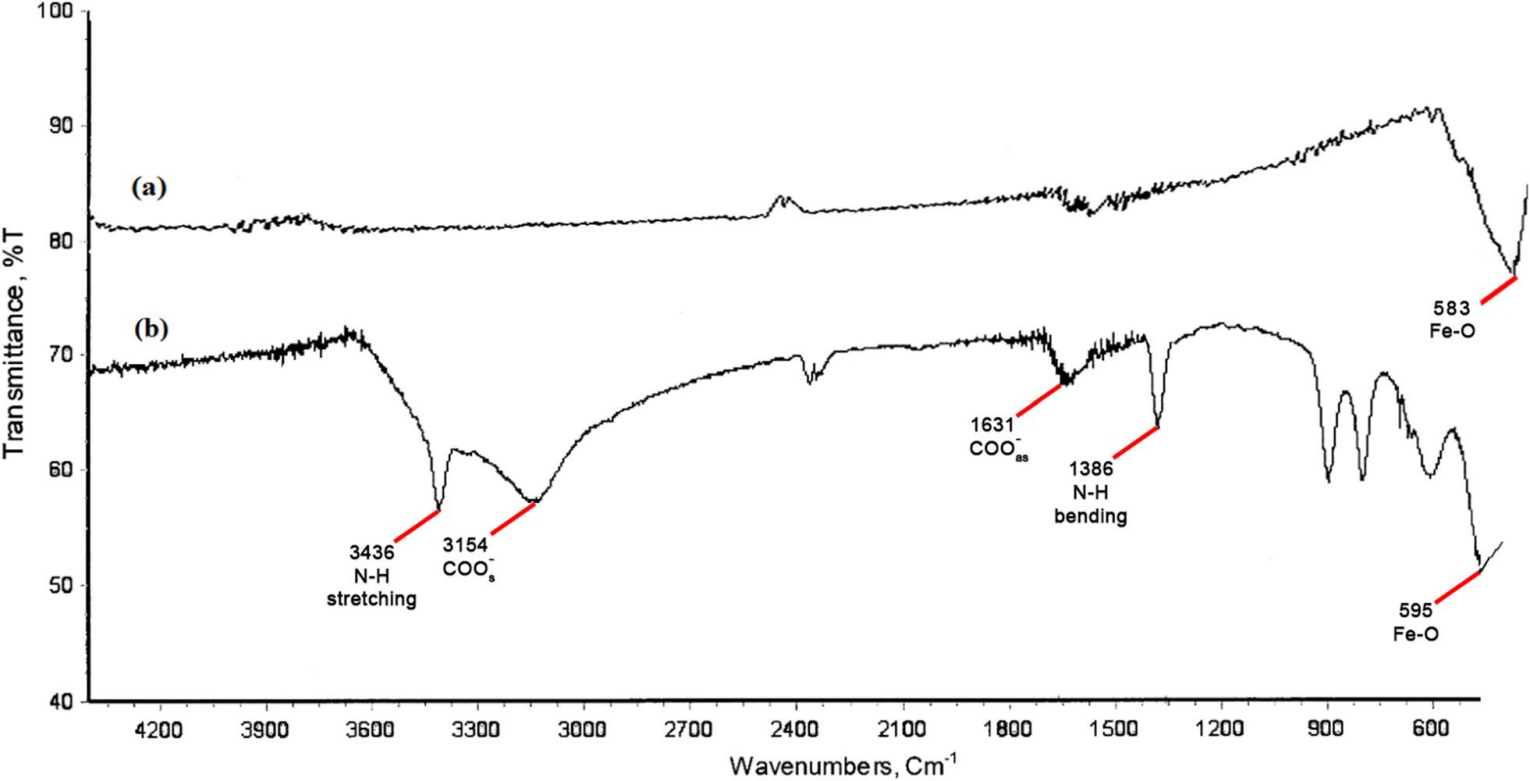
The comparative FT-IR spectra of the Fe_3_O_4_ (a) and Fe_3_O_4_@l-arginine (b)

The FT-IR spectra of the bare Fe_3_O_4_ and Fe_3_O_4_@l-arginine nanocomposite are presented in Fig. 4. Bare magnetite nanoparticles are easily distinguished by strong absorption peaks at 583 cm^−1^ because of the stretching vibration of the Fe–O band (Fig. 4a). Figure 4b shows the FT-IR spectrum of Fe_3_O_4_@l-arginine nanocomposite. The existence of Fe_3_O_4_ NPs is determined by the strong adsorption band at 595 cm^−1^ related to the Fe–O vibrations. In the case of Fe_3_O_4_@l-arginine, the additional adsorption peaks at 1386, 1631 and 3154, 3436 cm^−1^ are due to bending vibration of N–H, asymmetric and symmetric stretching vibrations of COO^−^, and stretching vibrations of N–H, respectively, which indicate the presence of bonded arginine on the surface of magnetite nanoparticles. Furthermore, the connections and interactions between COO^−^ groups and metal atoms are completely according to pervious literature [45–48].

**Fig. 5:**
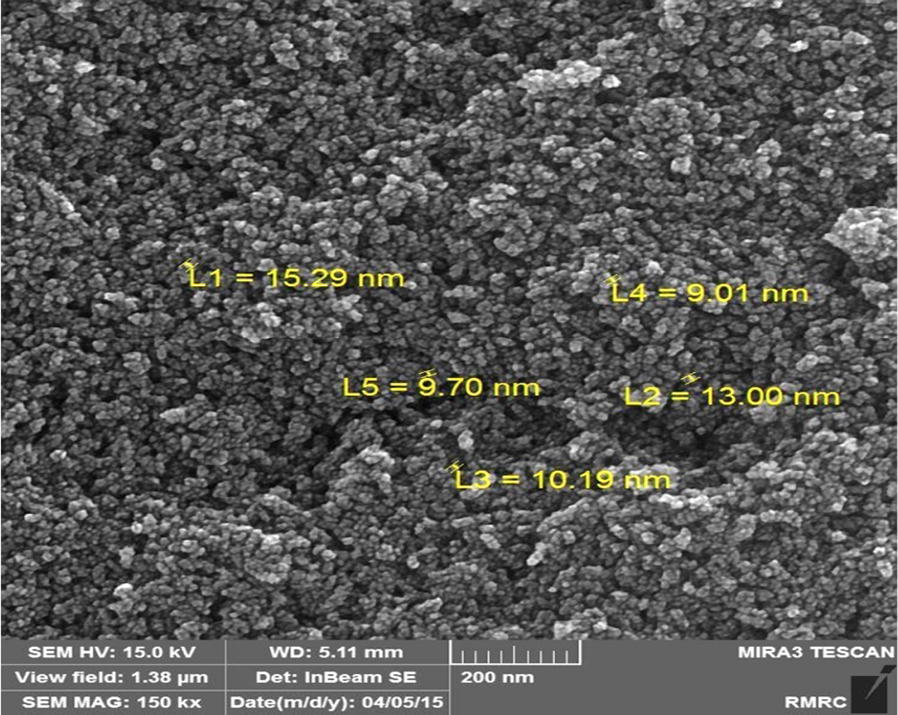
SEM image of the Fe_3_O_4_ @l-arginine nanocomposite

In order to investigate the morphology and particle size of nanoparticles, SEM image of the mesoporous is illustrated in Fig. 5. The SEM image of the magnetite nanoparticles modified with arginine indicate spherical shape with an average diameter about 10–15 nm.

**Fig. 6:**
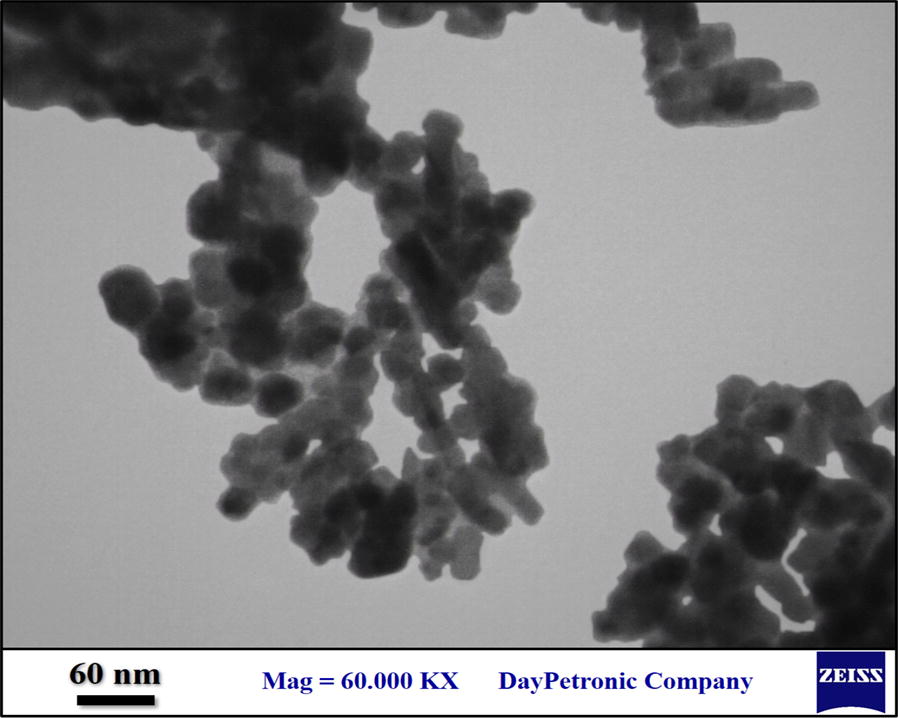
TEM image of Fe_3_O_4_@l-arginine

The morphology and particle size of Fe_3_O_4_@l-arginine were investigated using transmission electron microscopy (TEM) (Fig. 6). The TEM image of this nanocomposite shows that the average particle size of Fe_3_O_4_@l-arginine is around 10–20 nm which was confirmed by the SEM image.

## Discussions

**Fig. 7:**
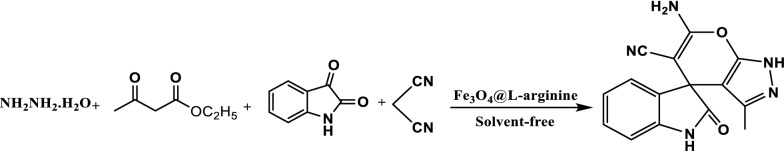
The model reaction for the synthesis of spiro[indoline-3,4′- pyrano[2,3-c]pyrazole]

Initially, to obtain the best reaction conditions, we selected reaction of hydrazine, ethyl acetoacetate, isatin and malononitrile as model reaction. Different catalysts, solvents and temperatures were examined in the four-component preparation of spiro[indoline-3,4′- pyrano[2,3-c]pyrazole] (Fig. 7).

Firstly, the model study was performed in various solvents including EtOH, DMF, H_2_O, CH_3_CN and PhCH_3_ under reflux conditions and also under solvent-free conditions using Fe_3_O_4_@l-arginine nanocomposite. The summarized results of Table 1 show that the best results were obtained under solvent-free conditions. To further improve the yield and decrease the reaction time, we used the different reaction temperatures under solvent-free conditions. Further increase in temperature from room temperature to 80 °C in the model study did not have any remarkable influence on the reaction time and production yield (Table 1).

**Table 1:** The effect of solvents on the model reaction in the presence of Fe_3_O_4_@l-arginine

Entry	Solvent	Time (min)	Yield (%)^a^
1	EtOH (reflux)	70	60
2	DMF (reflux)	120	50
3	H_2_O (reflux)	130	35
4	CH_3_CN (reflux)	120	45
5	Toluene (reflux)	240	25
6	Solvent-free (r.t.)	60	96
7	Solvent-free (40 °C)	60	95
8	Solvent-free (80 °C)	60	96

Reaction conditions: hydrazine monohydrate, isatin, ethyl acetoacetate, malononitrile (molar ratio: 1:1: 1.2:1) using (0.01 g) of Fe_3_O_4_@l-arginine

^a^Isolated yields

Afterward, the model was performed using several catalysts including ZnO, CuI, MgO, Na_2_CO_3_, Et_3_N, piperidine, Fe_3_O_4_, CaO, SiO_2_, and Fe_3_O_4_@l-arginine under solvent-free conditions. As can be seen from Table 2, no product was afforded in the absence of a catalyst (Table 2, entry 1). Also, it is noticed that Fe_3_O_4_@l-arginine has a significant effect in the yield of the corresponding product and reaction time (Table 2, entry 11). Next, various amounts of the Fe_3_O_4_@l-arginine were used in the model reaction. As shown in Table 2, the best experimental operation conditions included 8 mol % of the Fe_3_O_4_@l-arginine. With increasing the amount of nanocatalyst, no considerable change was observed in the product yield and reaction time. In comparison, a decrease in the catalyst amount cause to decrease the product yield. Hence, 8 mol % Fe_3_O_4_@l-arginine was selected as the optimum amount in the model reaction (Table 2).

**Table 2:** The model study catalyzed in the presence of various catalysts

Entry	Catalyst	Catalyst loading (mol %)	Time (min)	Yield(%)^a^
1	None	**–**	120	0
2	ZnO	15	120	35
3	CuI	15	120	42
4	MgO	15	100	65
5	Na_2_CO_3_	15	80	72
6	Et_3_N	15	60	70
7	Piperidine	15	80	55
8	Fe_3_O_4_	15	120	40
9	CaO	15	80	65
10	SiO_2_	15	120	35
11	Fe_3_O_4_@l-arginine	15	60	96
12	Fe_3_O_4_@l-arginine	10	60	96
13	Fe_3_O_4_@l-arginine	8	60	96
14	Fe_3_O_4_@l-arginine	5	90	70

Reaction conditions: hydrazine monohydrate (1 mmol), isatin (1 mmol), ethyl acetoacetate (1 mmol) and malononitrile (1 mmol) under solvent-free at room temperature

^a^Isolated yields

**Fig. 8:**
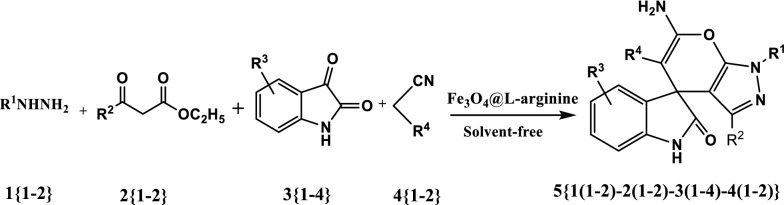
Synthesis of spiro[indoline-3,4′-pyrano[2,3-c]pyrazole] derivatives

**Fig. 9:**
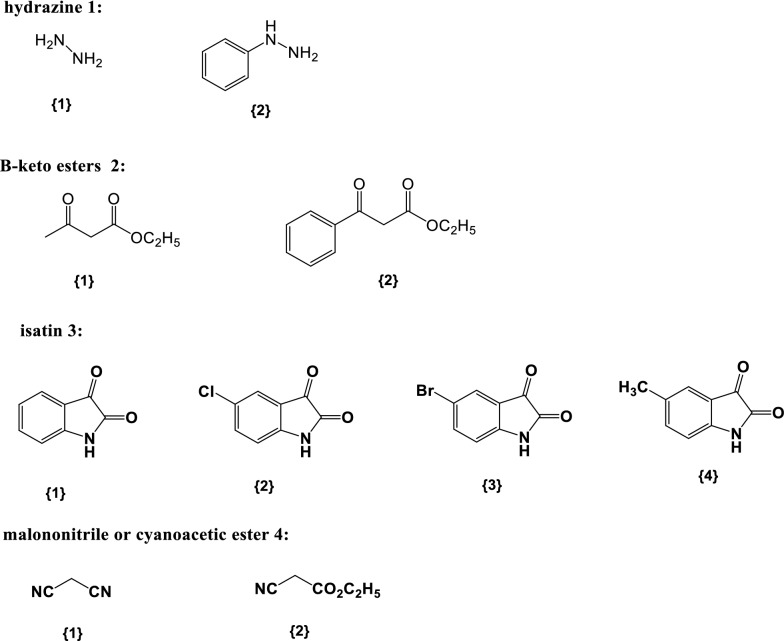
Diversity of the reagents

The optimized reaction conditions were tested for library constructions with two hydrazines 1{1–2}, β-keto esters 2{1–2}, four isatins 3{1–4}, and two acetonitrile derivatives 4{1–2} (Figs. 8 and 9).

The corresponding spiro-[indoline-3,4′-pyrano [2,3-c]pyrazole] derivatives 5 were obtained in good yields at room temperature under solvent-free conditions (Table 3). The protocol was effective with isatins containing either electron-withdrawing (halides) or electron-donating (alkyl) groups.

**Table 3:** Synthesis of spiro[indoline-3,4′- pyrano[2,3-c]pyrazole] derivatives using Fe_3_O_4_@l-arginine under solvent-free conditions

Entry	Product	Yield (%)^a^	M.p. °C	Lit. M.p. °C
1	5{1,1,1,1}	96	286–288	285–286 [18]
2	5{1,1,2,1}	91	297–298	297–298 [18]
3	5{1,1,3,1}	95	283–285	282–283 [18]
4	5{2,1,1,1}	90	225–227	227–229 [18]
5	5{1,2,3,1}	91	257–259	256–257 [18]
6	5{1,2,1,1}	90	282–284	280–281 [18]
7	5{1,2,1,2}	87	240–242	242–243 [18]
8	5{1,2,3,2}	89	256–258	257–259 [18]
9	5{1,2,2,2}	85	266–268	265–267 [18]
10	5{1,1,4,1}	94	278–280	279–281 [18]
11	5{1,2,4,1}	86	246–248	247–249 [18]
12	5{1,2,4,2}	87	262–264	260–263 [18]
13	5{2,1,4,1}	89	220–222	222–224 [18]
14	5{2,1,3,2}	92	212–214	–^b^
15	5{2,1,2,2}	90	279–281	–^b^
16	5{2,1,4,2}	97	198–200	–^b^

^a^Isolated yield

^b^New Products

The model study was run several times using recycled magnetic nanocomposite to consider the recoverability level and lifetime of the Fe_3_O_4_@l-arginine nanocomposite. The results showed that the recovered magnetic nanocomposite can be utilized for five successive runs with a negligible decrease in its activity (Table 4).

**Table 4:** The catalyst reusability for the synthesis of spiro[indoline-3,4′-pyrano[2,3-c]pyrazole]

Cycle	First	Second	Third	Fourth	Fifth
Yield (%)^a^	96	94	93	89	88

^a^Yields refer to the isolated pure product

**Fig. 10:**
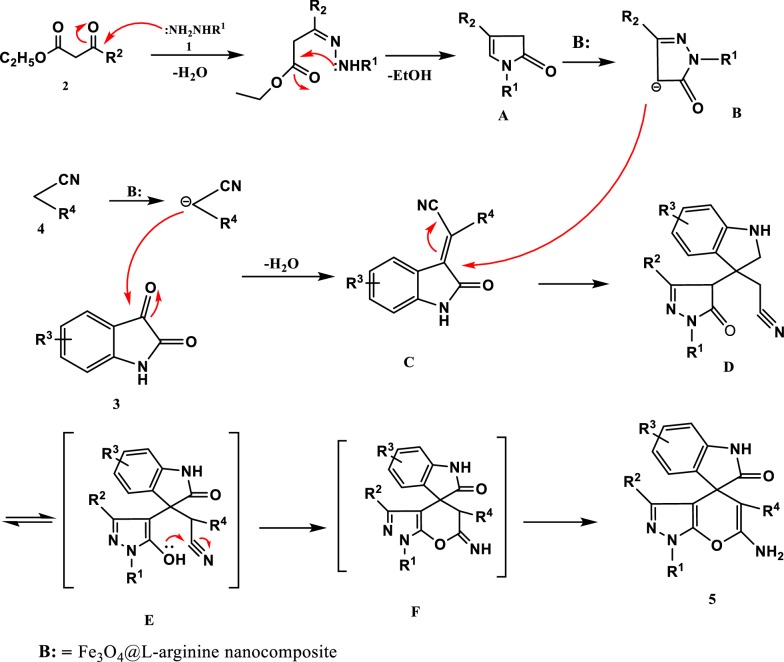
The proposed mechanism for the synthesis of spiro[indoline-3,4′-pyrano[2,3-c]pyrazoles] catalyzed by Fe_3_O_4_@l-arginine nanocomposite

A possible mechanism for the synthesis of spiro[indoline-3,4′-pyrano[2,3-c]pyrazoles] using Fe_3_O_4_@l-arginine MNPs is presented in Fig. 10. This mechanism is based on the results of our experiment and some literature [19]. According to this mechanism, a condensation of hydrazine 1 with β-keto esters 2 is offered to give the intermediate **A**. Next, A Knoevenagel condensation of isatin **3** with malononitrile or ethyl cyanoacetate **4** is presented to provide the intermediate **C**. Next, the Michael addition of the intermediate **A** to **C** catalyzed by Fe_3_O_4_@l-arginine which provided the intermediate **D**. Then, the intermediate **F** was prepared via the intramolecular cyclization of intermediate **D**. Eventually, the intermediate **F** is tautomerized to product **5** (Fig. 10).

## Conclusions

In conclusion, we have demonstrated that Fe_3_O_4_@l-arginine nanocomposite is an effective catalyst for the MCRs of hydrazine, β-keto esters, isatins, and malononitrile or ethyl cyanoacetate under solvent-free conditions at room temperature. The heterocyclic compounds including spiro[indoline-3,4′-pyrano[2,3-c]pyrazole] derivatives were obtained in high yields. The catalyst can be recovered and reused at least up to five runs for the synthesis of corresponding product. The one-pot nature and the use of heterogeneous solid Brønsted basic catalyst as an eco-friendly structure make it an interesting alternative to multi-step approaches.

## Experimental section

### Chemicals and apparatus

Chemicals were purchased from the Sigma-Aldrich and Merck in high purity. All of the materials were of commercial reagent grade and were used without further purification. The synthesis and characterization of the studied compounds were previously reported [49]. Melting points of products were determined by Electro thermal 9200. ^1^H NMR and ^13^C NMR spectra were obtained on Bruker 400 MHz spectrometer with DMSO-*d*_6_ as solvent using TMS as an internal standard. FT-IR spectrum was recorded on Magna-IR, spectrometer 550. The elemental analyses (C, H, N) were obtained from a Carlo ERBA Model EA 1108 analyzer. Powder X-ray diffraction (XRD) was carried out on a Philips diffractometer of X’pert Company with mono chromatized Cu Kα radiation (λ = 1.5406 Å). Microscopic morphology of products was visualized by SEM (LEO 1455VP). The compositional analysis was done by energy dispersive analysis of X-ray (EDX, Kevex, Delta Class I). Transmission electron microscopy (TEM) was performed with a Jeol JEM-2100UHR, operated at 200 kV.

### Preparation of Fe_3_O_4_@l-arginine nanocomposite

**Fig. 11:**
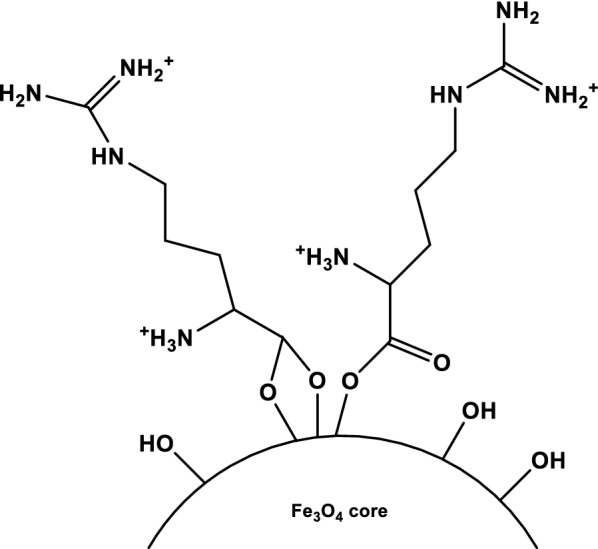
Possible structure for l-arginine coating on Fe_3_O_4_ nanoparticles, the inset is showing the molecular structure of Fe_3_O_4_

Fe_3_O_4_@l-arginine was prepared according to previous report in the literature with some modifications [50]. In a typical experiment, FeCl_3_·6H_2_O (13 g, 0.048 mol), FeCl_2_·4H_2_O (4.8 g, 0.024 mol) and arginine (16.7 g, 0.096 mol) were dissolved in 100 mL deionized water. Then, the solution pH was adjusted to 11 with NaOH solution (2 M) to form a black suspension. Next, the reaction mixture was reflux under Ar atmosphere for 12 h. Finally, the prepared nanocomposite was separated from the reaction media by an external magnet and washed several times with deionized water and dried in an oven overnight to yield Fe_3_O_4_@l-arginine (Fig. 11).

### General procedure for the synthesis of spiro[indoline-3,4′-pyrano[2,3-c]pyrazole]

Fe_3_O_4_@l-arginine (8 mol %) was added to a solution of hydrazine (1 mmol), β-keto esters (1 mmol), isatin derivatives (1 mmol), and malononitrile/ethyl cyanoacetate (0.06 g, 1 mmol). The reaction mixture was stirred under solvent-free conditions at room temperature for the appropriate times. After completion of the reaction [as determined by thin layer chromatography (TLC)], the reaction mixture was dissolved in dichloromethane and the catalyst was separated magnetically. The solvent was evaporated and the residue was recrystallized from ethanol to afford the pure product.

All of the products were characterized and identified with m.p., ^1^H NMR, ^13^C NMR and FT-IR spectroscopy techniques. Spectral data of the new products are given below.

### Spectral data of new compounds

#### Ethyl-6′-amino-5-bromo-3′-methyl-2-oxo-1′-phenyl-1′*H*-spiro[indoline-3,4′-pyrano[2,3-c]pyrazole]-5′-carboxylate (5{2,1,3,2})

White solid, m.p. 212–214 °C. IR (KBr) (ν_max_/cm^−1^): 3396 (NH_2_), 3143 (NH), 1714 (CO); ^1^H NMR (400 MHz, DMSO-*d*_6_) δ: 0.92 (t, 3H, *J *= 7.6, CH_3_CH_2_OCO), 1.23 (s, 3H, CH_3_), 3.70 (q, 2H, *J *= 7.8, CH_3_CH_2_OCO), 6.93 (d, 1H, *J *= 7.6, ArH(isatin)), 7.34 (d, 1H, *J *= 7.4, ArH (isatin)), 7.51–7.58 (m, 3H, ArH(PhNHNH_2_)), 7.61 (s, 2H, NH_2_), 7.74 (s, 1H, ArH(isatin)), 7.78 (d, 2H, *J *= 7.8, ArH (PhNHNH_2_)), 10.90 (s, 1H, NH); ^13^C NMR (100 MHz, DMSO-*d*_6_) δ: 11.2, 14.8, 26.8, 57.2, 91.2, 113.1, 119.9, 124.5, 126.6, 129.1, 129.7, 131.2, 131.9, 135.2, 139.6, 148.7, 152.1, 161.8, 162.9, 171.2, 177.5; Anal. Calcd for C_23_H_19_BrN_4_O_4_ (Mr = 495.33) (%): C 55.77, H 3.87, N 11.31. Found (%): C 55.87, H 3.79, N 11.36.

#### Ethyl-6′-amino-5-chloro-3′-methyl-2-oxo-1′-phenyl-1′*H*-spiro[indoline-3,4′-pyrano[2,3-c]pyrazole]-5′-carboxylate (5{2,1,2,2})

White solid, m.p. 279–281 °C. IR (KBr) (ν_max_/cm^−1^): 3377 (NH_2_), 3185(NH), 1712 (CO); ^1^H NMR (400 MHz, DMSO-*d*_6_) δ: 1.09 (t, 3H, *J *= 7.8, CH_3_CH_2_OCO), 1.24 (s, 3H, CH_3_), 3.72 (q, 2H, *J *= 7.6, CH_3_CH_2_OCO), 6.82 (d, 1H, *J *= 7.4, ArH(isatin)), 7.21 (d, J = 7.6, ArH(isatin)), 7.48–7.53 (m, 3H, *J *= 7.7 ArH(PhNHNH_2_)), 7.68 (s, 2H, NH_2_), 7.72 (s, 1H, ArH(isatin)), 7.81 (d, 2H, *J *= 7.8 ArH (PhNHNH_2_)), 10.93 (s, 1H, NH); ^13^C NMR (100 MHz, DMSO-*d*_6_) δ: 10.8, 15.1, 23.9, 55.2, 93.2, 113.1, 120.1, 123.5, 125.1, 128.7, 129.9, 132.1, 133.1, 135.1, 140.2, 147.1, 151.4, 162.4, 162.9, 174.1, 178.1; Anal. Calcd for C_23_H_19_ClN_4_O_4_ (Mr = 450.88) (%): C 61.27, H 4.25, N 12.43. Found (%): C 61.20, H 4.33, N 12.39.

#### Ethyl-6′-amino-3′,5-dimethyl-2-oxo-1′-phenyl-1′*H*-spiro[indoline-3,4′-pyrano[2,3-c]pyrazole]-5′-carboxylate (5{2,1,4,2})

White solid, m.p 198–200 °C. IR (KBr) (ν_max_/cm^−1^): 3370 (NH_2_), 3181 (NH), 1709 (CO); ^1^H NMR (400 MHz, DMSO-*d*_6_) δ: 1.13 (t, 3H, *J *= 7.6, CH_3_CH_2_OCO), 1.22 (s, 3H, CH_3_), 2.25 (s, 3H, CH_3_), 3.78 (q, 2H, *J *= 7.7, CH_3_CH_2_OCO), 7.08 (d, 1H, 2H, *J *= 7.8, ArH (isatin)), 7.16 (d, 1H, 2H, *J *= 7.6, ArH (isatin)), 7.34–7.41 (m, 3H, *m*, *p*, ArH (PhNHNH_2_)), 7.45 (s, 1H, ArH (isatin)), 7.66 (s, 2H, NH_2_), 7.68 (d, 2H, 2H, *J *= 7.6, ArH (PhNHNH_2_)), 10.78 (s, 1H, NH); ^13^C NMR (100 MHz, DMSO-*d*_6_) δ: 11.6, 14.8, 21.4, 25.7, 56.1, 92.7, 115.4, 117.9, 121.2, 123.4, 125.8, 126.9, 128.6, 129.1, 129.9, 137.1, 147.1, 148.3, 159.4, 162.6, 173.2, 176.8; Anal. Calcd for C_24_H_22_N_4_O_4_ (Mr = 430.46) (%): C 66.97, H 5.15, N 13.02. Found (%): C 66.91, H 5.19, N 12.97.

## Data Availability

The datasets generated and analyzed during the current study available from the corresponding author on reasonable request.

## Abbreviations

MCR: multi-component reactions
EtOH: ethanol
SEM: scanning electron microscope
TEM: transmission electron microscopy
FT-IR: Fourier transform infrared spectroscopy
XRD: powder X-ray diffraction
EDX: energy dispersive analysis of X-ray
NMR: Nuclear Magnetic Resonance
TLC: thin layer chromatography
